# Symptom Burden and Mobility in Older Adults: A Qualitative Study to Inform Clinical Innovation

**DOI:** 10.1093/geroni/igaf122.3662

**Published:** 2025-12-31

**Authors:** Michelle McKay, Tasha Martin-Peters, Kerry Shields, Rebekah Deroo, Christina Whitehouse

**Affiliations:** Villanova University, Norristown, Pennsylvania, United States; Villanova University, Villanova, Pennsylvania, United States; Villanova University, Villanova, Pennsylvania, United States; Villanova University, Villanova, Pennsylvania, United States; Villanova University, Villanova, Pennsylvania, United States

## Abstract

Approximately 17.6 million adults aged ≥65 years have mobility limitations, which may include the inability to walk a quarter of a mile or climb stairs independently. Mobility limitations are the most common limitation among older adults. Limitations in mobility can lead to disability, decreased quality of life, and poor health outcomes. Older adults report a variety of symptoms that contribute to their mobility limitations, which impact their participation in activities of daily living. There is a lack of data regarding the assessment and management of the symptom burden experienced by older adults. The objective of this study was to explore the perspectives of nurse practitioners (NPs) in identifying and managing the symptom burden associated with mobility limitations in community-dwelling older adults. We conducted 14 semi-structured interviews of NPs in primary care settings. Interviews were professionally transcribed and analyzed using thematic analysis. Six themes were identified: 1) symptom burden over disease labels; 2) functional status and daily living; 3) interprofessional and family involvement; 4) prevention of hospitalization and functional decline; 5) motivation and what matters most; and 6) conservative and holistic treatment approaches. The results of this study provided insight on healthcare provider’s assessment and management of symptoms responsible for mobility limitations in community-dwelling older adults. These results will support the development of a symptom burden risk assessment tool and innovative interventions focused on symptom management to improve mobility and decrease the associated negative outcomes.

